# Nurse led, primary care based antiretroviral treatment versus hospital care: a controlled prospective study in Swaziland

**DOI:** 10.1186/1472-6963-10-229

**Published:** 2010-08-05

**Authors:** Ciaran P Humphreys, John Wright, John Walley, Canaan T Mamvura, Kerry A Bailey, Sweetness N Ntshalintshali, Robert M West, Aby Philip

**Affiliations:** 1 Public Health Wales, Carmarthen, UK; 2 Bradford Institute for Health Research, Bradford Royal Infirmary, Leeds, UK; 3 Nuffield Centre for International Health and Development, Leeds Institute of Health Sciences, University of Leeds, Leeds, UK; 4 Good Shepherd Hospital, Siteki, Swaziland

## Abstract

**Background:**

Antiretroviral treatment services delivered in hospital settings in Africa increasingly lack capacity to meet demand and are difficult to access by patients. We evaluate the effectiveness of nurse led primary care based antiretroviral treatment by comparison with usual hospital care in a typical rural sub Saharan African setting.

**Methods:**

We undertook a prospective, controlled evaluation of planned service change in Lubombo, Swaziland. Clinically stable adults with a CD4 count > 100 and on antiretroviral treatment for at least four weeks at the district hospital were assigned to either nurse led primary care based antiretroviral treatment care or usual hospital care. Assignment depended on the location of the nearest primary care clinic. The main outcome measures were clinic attendance and patient experience.

**Results:**

Those receiving primary care based treatment were less likely to miss an appointment compared with those continuing to receive hospital care (RR 0·37, *p *< 0·0001). Average travel cost was half that of those receiving hospital care (*p *= 0·001). Those receiving primary care based, nurse led care were more likely to be satisfied in the ability of staff to manage their condition (RR 1·23, *p *= 0·003). There was no significant difference in loss to follow-up or other health related outcomes in modified intention to treat analysis. Multilevel, multivariable regression identified little inter-cluster variation.

**Conclusions:**

Clinic attendance and patient experience are better with nurse led primary care based antiretroviral treatment care than with hospital care; health related outcomes appear equally good. This evidence supports efforts of the WHO to scale-up universal access to antiretroviral treatment in sub Saharan Africa.

## Background

Antiretroviral treatment (ART) has become increasingly available to patients with HIV/AIDS in sub Saharan Africa. The unfamiliarity of these new and potentially toxic drugs has, for the most part, limited their use to hospital-based, secondary care specialist clinics.

Secondary care can be difficult to access for poor rural populations and, in many countries, lacks the capacity to cope with the expanding numbers of patients on ART follow-up care. The ultimate goal of many national ART programmes is, therefore, to promote primary care and community based ART.

There are an estimated 2·7 million new HIV infections globally each year. Despite rapid progress, the universal access goals remain far off. In low- and middle-income countries in 2007 only 31% of people estimated to be in need of treatment were receiving it [1]. Achieving universal access on such a major scale will depend on shifting skills from hospital to primary care settings.

Primary care and community based ART programmes have been implemented in highly resourced and research contexts [2,3]. The safety and effectiveness of such programmes in typical African district settings remain uncertain. The potential threat of ART drug resistance from poor adherence and monitoring in such programmes is a particular concern [4,5].

Modelling has suggested the potential of universal yearly HIV counselling and testing (HCT) and immediate ART, as a means to eliminate HIV [6]. The safety and effectiveness of primary care clinic nurse and lay worker HCT and ART follow-up care is critical to the feasibility of such an approach [7].

This study reports the results of a prospective, controlled evaluation of nurse led, primary care based ART in a rural African district on attendance and health outcomes.

## Methods

### Setting

Lubombo is a predominantly rural region of Swaziland. It has a population of 250,000 and one of the highest prevalence rates of HIV in the world with 26% of those aged 15-44 infected with HIV [8]. The region has one district general hospital, two health centres and a network of 30 nurse-run primary care clinics.

### Study design

An evaluation of planned service change was undertaken with a prospective cohort and control group comparison. A phased approach was taken following recommendations for evaluations of complex interventions [9,10]. A piloting phase was undertaken May to December 2006 to assess feasibility and early implementation. The study reported on here followed refinement of the protocol and wider testing, with recruitment from January 2007 to June 2007 and follow-up until November 2007.

### Study population

Adults (aged over 14) on ART for at least four weeks who had a CD4 count over 100 were included in the study once they were assessed as clinically suitable for nurse led follow-up by a medical officer. Children were not included within the evaluation as more detailed clinical and social assessment was undertaken before transfer to the primary care centre.

**Figure 1:**
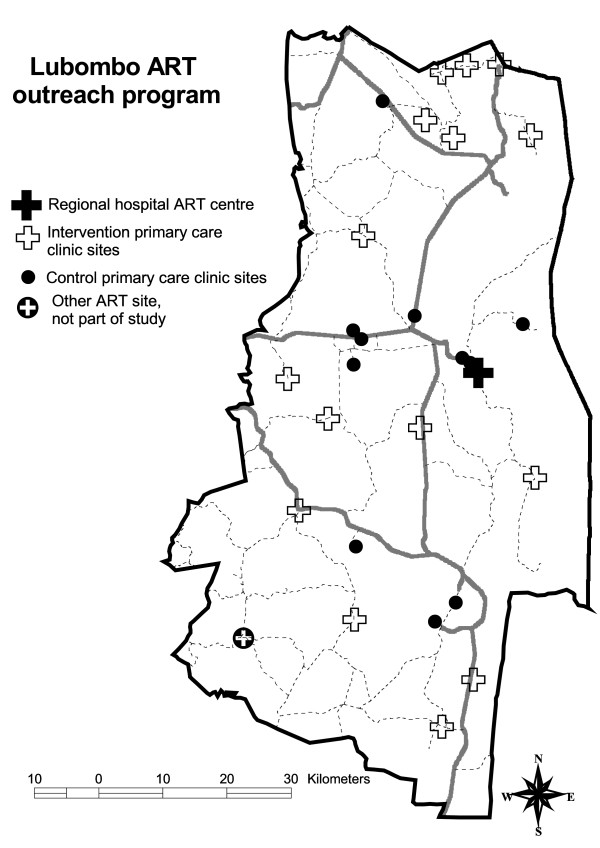
**Map of clinics included in study**.

The intervention group included patients receiving ART who resided or worked within the catchment area of the intervention clinics. The control population consisted of adult patients who resided within the catchment area of the control clinics (see Figure 1). Individuals who would otherwise be eligible for the study who died before their next scheduled appointment were not included in the analysis as they would have had no opportunity to take up the intervention.

### Selection of primary care sites

Primary care centres were purposively selected for involvement in the ART programme. For the pilot phase seven primary care centres in close geographical proximity and with good clinical links were selected to provide a favourable context for initial implementation. For the main study an additional eight primary care centres were selected on the basis of being most remote from the main hospital, providing a contrast to the pilot sites and increasing the representativeness of these sites. The control sites were the remaining eleven primary care clinics in the region with patients participating in the ART programme.

### Intervention

The primary care based ART programme consisted of an initial training programme for primary care centre nurses developed by the hospital team, followed by a monthly outreach support visit.

Patients attending the hospital ART programme who fulfilled the inclusion criteria were assigned to intervention or control groups depending on their nearest clinic. Those in the intervention group were then offered primary care centre follow up. Patients could continue treatment at the hospital if that was their preference.

An outreach team of at least one counsellor and nurse provided monthly visits to each of the primary care clinics. During these visits they undertook clinical review, CD4 count and other appropriate investigations, counselling, prescribing and dispensing of medications. Complications such as side effects of medication and opportunistic infections were managed at the clinic where possible. If required, patients were referred for further assessment and management at the main hospital site. Over the first seven months of the study primary care clinic nurses and lay support workers increasingly took over ART review and ongoing adherence counselling, and the hospital staff role became to supervise and assist. During the study six clinics essentially managed their own patients, whereas nine clinics continued to rely on the hospital team for much of the clinical care.

### Outcome measures

The primary outcome measure was clinic attendance. Clinic attendance was treated as a binary outcome: the proportion of individuals attending all scheduled clinic appointments was compared with the proportion of individuals failing to attend at least one scheduled clinic appointment. Arriving after the date of the scheduled appointment was considered a failure to attend. Secondary outcomes included patient experience, loss to follow-up (failing to attend a scheduled clinic appointment for over 90 days; loss to follow-up was not considered as missing an attendance under the primary outcome), and change in health related outcomes (CD4 count, weight, and death). Change in CD4 count and weight were assessed by comparing the most recent CD4 and weight before enrolment in the study to the last measured during the study.

### Data collection and analysis

Patient outcomes were collected through the computer based national ART monitoring and evaluation system. This system is the key administrative database for scheduling clinic attendances and ensuring patient records are available on the correct clinic day and site.

Outcomes among those in the intervention group were compared with outcomes among those in the control group. Separate analyses were undertaken for those who took up the intervention offered (per protocol) and all those offered the intervention irrespective of whether they took it up (modified intention to treat). Bivariate analysis was undertaken using the Chi square test, Fisher's exact test, two tailed t-test for comparison of means or Kruskal-Wallis test as appropriate. The analysis plan included multilevel multivariable logistic regression modelling to provide an estimate of the clustering effect: patients clustered within health centres; and that of other variables. Modelling was undertaken for the primary outcome, clinic attendance, and included sex, age, clinical stage on initiation of ART, most recent weight at start of the study and whether or not an individual was within the intervention or control group. This model was programmed in Stata version 10 (StataCorp 2007).

### Sample size

Assuming an individual had a probability of missing an appointment during the period of follow-up of 0·3 we wished to detect a reduction to 0·15 with intervention. Given a ratio of intervention to control of 2:1, alpha = 0.05, power = 80% yields a sample size of 103 in the control and 206 in the intervention groups. Assuming an average of 15 patients per primary care centre then a rough approximation of design factor with intracluster coefficient = 0·01 is 1·14 yielding a sample size of 118 controls and 235 intervention groups.

### Assessment of patient experience

Structured interviews were carried out between July and August 2007 to assess satisfaction with services. Patients enrolled into intervention or control groups by the 1^st ^April 2007 were randomly selected for interview. The intervention group was stratified by clinic site before randomisation to ensure appropriate representation from each site. Interviews were undertaken by two trained interviewers for whom siSwati was their native language and who were not hospital staff. Satisfaction was rated using a Likert scale.

### Ethics

Ethical approval was obtained from the Leeds University Faculty of Medicine and Health Research Ethics Committee. The study was undertaken with agreement from the Swaziland Ministry of Health. This was an evaluation of planned service change. Consent for transfer to nurse led primary care based antiretroviral treatment was sought from those in the intervention group. Consent was sought verbally by the assessing physician and recorded by the physician in a written proforma. No personally identifiable data was shared beyond the clinical team.

## Results

**Figure 2:**
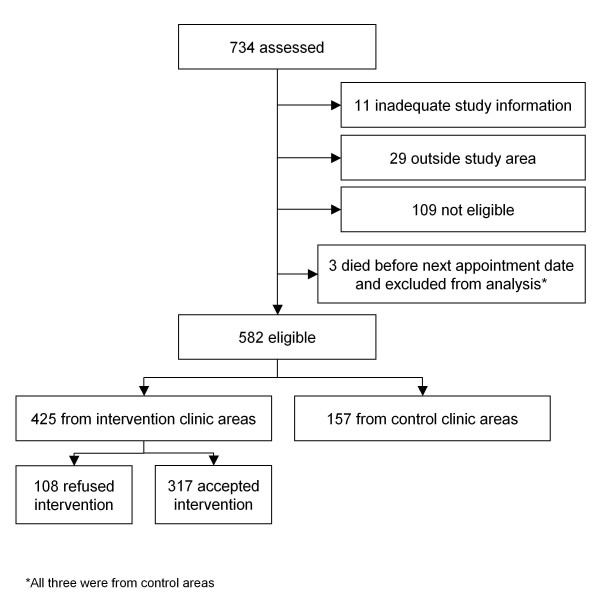
**Flow diagram of eligibility for study**.

582 (80%) of the 734 individuals assessed for the study were eligible (Figure 2). A quarter of those offered the intervention refused. The most common reasons for refusal were concern over stigma (23% of men; 37% of women) and not convenient due to work location (50% of men; 16% of women); other reasons included not convenient due to home location; preference for hospital; and the patient receiving other medical treatment at the hospital site.

Participants in intervention and control groups were similar at the start of the study in terms of age, gender, mean CD4 count, mean weight and proportion with stage IV disease. However, the mean time on ART on entering the study was greater in the control group (Table 1). The average length of time in the study was 25 days longer for the intervention group (267 days) than the control group (242 days, *p *< 0.001). Similarly the average number of clinic attendances after recruitment was greater in the intervention group (7.1) than the control group (6.1, *p *< 0.001).

**Table 1:** Characteristics of participants

	Intervention (317)	Control (157)	*P *value
Female (%, *n*)	67% (212)	68% (106)	0.9

Age (years, mean, standard deviation)	39.3 (10.9)	40.0 (11.8)	0.7

CD4 (most recent, mean, standard deviation)	373 (204)	407 (206)	0.2

Weight (kg, most recent, mean, standard deviation)	62.7 (10.9)	64.8 (12.4)	0.07

Stage IV at start of ART (%, *n*)	20% (64)	22% (35)	0.6

Time on ART at start (days, mean, standard deviation)	347 (278)	506 (334)	< 0.0001

Length of follow-up (days, mean, standard deviation)	267 (48)	242 (53)	< 0.0001

Those in the intervention group were less likely to miss an appointment; but showed no significant difference in proportion lost to follow-up. Although there were significantly fewer deaths in the intervention group, this effect was not seen when those who refused intervention were included (modified intention to treat analysis; Table 2). There was no significant difference in other health outcomes measured (Table 3). The time between measurements was longer for the control group than the intervention group for weight (244 and 234 days respectively, *p *= 0.02) but not significantly different for CD4 count (359 and 340 days respectively, *p *= 0.2).

**Table 2:** Key outcome measures

	Intervention group		Control group		Relative risk	95% confidence intervals	*P *value
				
	*n*	%	*n*	%			
Per protocol analysis							

Any missed appointment	33	10·4	44	28	0·37	(0·25 to 0·56)	< 0·0001

Loss to follow-up	9	2·8	2	1·3	2·23	(0·49 to 10·19)	0·4

Died	0	0	4	2·5	0	(undefined)	0·01

Modified intention to treat analysis							

Any missed appointment	50	11·8	44	28	0·42	(0·29 to 0·60)	< 0·0001

Loss to follow-up	10	2·4	2	1·3	1·85	(0·41 to 8·34)	0·5

Died	4	0·9	4	2·5	0·68	(0·34 to 1·37)	0·2

**Table 3:** Other outcome measures

	Valid result				Change in measure		
	
	Intervention		Control				
				
	*n*	%	*n*	%	Intervention	Control	*P *value
Per protocol analysis							

Weight change (kg, mean)	314	99%	156	99%	1·18	1·04	0·8

CD4 change (cells per microlitre; mean)	122	38%	56	36%	103	85	0·7

Modified intention to treat analysis							

Weight change (kg, mean)	419	99%	156	99%	1·09	1·04	0·9

CD4 change (cells per microlitre; mean)	122	38%	56	36%	101	85	0·6

### Multilevel, multivariable modelling

In fitting a multilevel logistic regression of 'any missed appointment' on all covariates there was very little variation at the primary care clinic level (*p *= 0·47). Being in the intervention, rather than the control group, was the only variable significantly associated with clinic attendance in this model (odds ratio 0.3, 95% confidence intervals 0.18-0.49, *p *< 0.0005).

### Patient experience

No individual refused to participate in the study. Some of those selected for interview were not included as either the individual did not attend on the day of their scheduled appointment or, in a small number of cases, the individual had left the department before being identified by the interviewers. Of those initially selected for interview 74% (45) in the intervention group and 79% (44) in the control group underwent interview.

The average age was 36·8 for the intervention group and 39·2 for the control group (*p *= 0·18) and 26% of the intervention group were male, as compared with 36% of the control group (*p *= 0·31). Fewer of those from the intervention group had obtained high school education (*p *= 0·002).

### Cost of travel

In the intervention group the average cost of return transport for follow up care (0·74US$) was half that of those in the control group attending hospital (1·5US$), *p *= 0·001. Fifty-three per cent of those attending the intervention clinic said that the cost of travel was reduced by the intervention.

### Satisfaction with services

Most patients from both intervention and control groups were satisfied with most aspects of service (Table 4). Levels of satisfaction were higher among the intervention group for all aspects of satisfaction measured; however, this was only significant for satisfaction in the ability of staff to manage the patient's condition. After adjusting for education there was no significant difference in satisfaction (*p *= 0·96).

**Table 4:** Proportion satisfied or various satisfied with aspects of clinic and service.

	Intervention % (*n*)	Control % (*n*)	RR	95% CI	*P *value
Environment such as space, comfort at the clinic*	79·5% (35)	73·8% (31)	1·08	0·85-1·36	0·52

Ability of the staff to manage your condition^†^	100% (44)	81·4% (35)	1·23	1·06-1·42	0·003

That your confidentiality will be maintained by clinic staff	77.8% (35)	77·3% (34)	1·01	0·80-1·26	0·95

Confidentiality will not be breached by other people seeing you at clinic^‡^	31·8% (14)	18.2% (8)	1·75	0·82-3·75	0·14

Overall service in this clinic	82·2% (37)	65·9% (29)	1·25	0·97-1·61	0·08

*3 non responses; ^† ^2 non-responses; ^‡ ^1 non-response

Those attending the intervention clinics were asked how satisfied they were with attending the intervention clinic rather than the main hospital site for ART (all had previously attended the main hospital site for ART in accordance with the protocol). Of those providing a response (31; 69%): 81% (25) were very satisfied, 13% (4) were satisfied, 3% (1) were dissatisfied and 3% (1) were very dissatisfied.

When asked the reason for their level of satisfaction attending the intervention clinic rather than the main hospital site 72% (21) of those satisfied or very satisfied volunteered reduced cost as a reason. Other reasons given included being nearer to home, a shorter queue, being treated better by staff, receiving better care and that they would not be talked about at the intervention clinic. The two individuals dissatisfied/very dissatisfied cited as their reason the lack of a doctor at the intervention clinic saying they did not have the money to attend the main clinic, and delay because the team from the hospital arrived late at the intervention clinic, respectively.

## Discussion and conclusions

This service evaluation demonstrated outcomes from a nurse led primary care setting that are as good as or better than in the traditional hospital setting. International guidance has encouraged a public health approach to HIV care, and in particular decentralising HIV services into the community and integrating HIV prevention, treatment and care services within primary care in generalised epidemics [11]. There has been little evidence to support the efficacy or safety of this approach. This study demonstrated providing ART in the primary care setting reduces patient cost and increases attendance at scheduled appointments. There is a common concern by health staff that patients would fear a lack of competence or confidentiality at their local nurse led primary care centre. However, patients demonstrate high levels of satisfaction with services in the primary care setting. In spite of the lack of diagnostics, such as radiology, or medical opinion available on demand, patients were more satisfied in the ability of staff to manage their condition in the local clinic setting than the hospital setting. The lower educational attainment of those interviewed in the intervention group, combined with the fact that those who refused the intervention could not be included may contribute to a higher satisfaction rating among the intervention group.

This pragmatic study integrated a controlled evaluation into planned service development and so provides evidence of how the intervention works in a real world rural Africa setting rather than in a heavily resourced research conditions [12].

Intervention and control groups were allocated purposively. Cluster randomised introduction of the intervention was considered politically not acceptable by health service managers. Control and intervention groups were similar in weight and CD4 counts. Viral loads were not available for comparison between the groups as the study relied on normal clinical facilities.

A minimum of one month of ART was required for consideration for inclusion in the study. However, many of the patients recruited had been on treatment much longer than this (347 or 506 days on average for intervention and control groups) when recruited to the study. Those in the intervention group were on ART for a shorter duration than the control group. The inclusion of patients in the earlier stage of treatment, when defaulting and morbidity is higher, would be expected to worsen study outcomes [13]; to the contrary, outcomes were as good or better in the intervention group.

The intervention group had a longer average period of follow-up in the study than the control group. Having a longer period in the study gives a greater potential for adverse outcomes, particularly missed appointments, loss to follow-up or deaths. However, again the outcomes were as good or better in the intervention group suggesting the effectiveness of the intervention may be underestimated.

A number of studies have demonstrated good outcomes from ART in primary care settings [3,14-18]. An ecological approach has also been taken, describing mortality in populations where primary care based ART has been introduced [19]. However, none of these studies used a suitable comparison group in order to assess the outcomes against standard care. Community or primary care based ART outcomes are likely to be incomparable with standard hospital based outcomes. Unlike community based therapy, hospital registers are likely to include patients admitted severely ill who commence ART as an inpatient.

While our study was not a randomised controlled trial, it did include prospective recruitment of patients who met stated physician-assessed criteria and a combination of patient-centred, clinical and service outcomes. This is the only such prospective controlled study of ART scale up in resource limited settings of which we are aware.

A quarter of those offered primary care based ART preferred to stay at the hospital. This was often due to convenience, e.g. due to work location; while others were concerned about the potential for stigmatisation through attendance for HIV treatment in their own community setting. When shifting care from hospital to primary care settings service planners should assess and cater for the wishes of a minority of patients who may wish to access treatment outside their own community.

This study had minimum requirements for CD4 count and time on treatment as well as clinical assessment of patients before referring for nurse led follow-up. There were very close links between the clinics and the hospital team throughout the study. Furthermore, the study did not evaluate more complex aspects of patient care such as drug toxicities, opportunistic infections or treatment failure. There is a continued need for ready access and appropriate referral to more specialised clinical care for those receiving nurse led primary care based ART.

This study provides supporting evidence for the WHO recommendations for decentralisation of HIV care [1]. Achieving universal access to ART will require the transfer of services from overloaded hospital services to primary care settings for the benefit of both patients and staff. This transfer will depend on the shift of skills and training from doctors to nurses, and some tasks to lay workers. As delivery of ART care shifts from hospital to primary care settings systems need to address staffing and resources at the primary care level. The potential impact on existing primary care services including capacity, motivation, recruitment of staff and other service priorities needs to be further assessed. Further research is required to explore the feasibility and effectiveness of initiation of ART in a typical African community setting.

Modelling by WHO has suggested the potential of universal yearly HIV counselling and testing (HCT) and immediate ART, as a means to eliminate HIV [6]. This study shows that primary care clinic nurse and lay worker HCT and ART follow-up care can be safe and effective, which is critical to the feasibility of such an approach [7].

## Competing interests

Ciaran P Humphreys, Canaan T Mamvura, Sweetness N Ntshalintshali, Kerry A Bailey and Aby Philip were all members of the team implementing service changes described in the study. The authors declare they have no other competing interests.

## Authors' contributions

CH, JW, JW, CM, SN and AP contributed to the study design, implementation and write up. RW contributed to analysis design and analysis. CH also contributed to analysis. KB contributed to study implementation, data collection and write-up. All authors read and approved the final manuscript.

## Pre-publication history

The pre-publication history for this paper can be accessed here:

http://www.biomedcentral.com/1472-6963/10/229/prepub
